# Measuring Sarcopenia Severity in Older Adults and the Value of Effective Interventions

**DOI:** 10.1007/s12603-018-1104-7

**Published:** 2018-09-18

**Authors:** Joanna P. MacEwan, T. M. Gill, K. Johnson, J. Doctor, J. Sullivan, J. Shim, D. P. Goldman

**Affiliations:** 1 Precision Health Economics, 11100 Santa Monica Blvd. Suite 500, Los Angeles, CA 90025 USA; 2 Yale School of Medicine, New Haven, USA; 3 Novartis Pharmaceuticals, East Hanover, USA; 4 USC Schaeffer Center for Health Policy and Economics, Los Angeles, USA

**Keywords:** Sarcopenia, burden, societal value, mobility impairment, frailty

## Abstract

**Objectives:**

Little is known about the severity and long-term health and economic consequences of sarcopenia. We developed a sarcopenia index to measure severity in older Americans and estimated the long-term societal benefits generated by effective interventions to mitigate severity.

**Design:**

Using a micro-simulation model, we quantified the potential societal value generated in the US in 2010–2040 by reductions in sarcopenia severity in older adults. All analyses were performed in Stata and SAS. Setting & Participants: Secondary data from the National Health and Nutrition Examination Survey (NHANES) (N = 1634) and Health and Retirement Study (HRS) (N = 952) were used to develop a sarcopenia severity index in older adults.

**Measurements:**

Multitrait multi-method and factor analyses were used to validate and calibrate the sarcopenia severity index, which was modeled as a function of gait speed, walking without an assistive device, and moderate physical activity.

**Results:**

In representative elderly populations, reducing sarcopenia severity by improving gait speed by 0.1 m/s in those with gait speed under 0.8 m/s generated a cumulative benefit of $65B by 2040 (2015 dollars). Improving walking ability in those with walking difficulty generated cumulative social benefit of $787B by 2040.

**Conclusions:**

Reducing sarcopenia severity would generate significant health and economic benefits to society— almost $800B in the most optimistic scenarios.

**Electronic Supplementary Material:**

Supplementary material is available for this article at 10.1007/s12603-018-1104-7 and is accessible for authorized users.

## Introduction

Sarcopenia, an age-related loss of muscle mass and strength (1, 2), contributes to disability and increases the risk of morbidity and mortality (3-8). Approximately one in four older adults (individuals over the age of 65) in the US has mobility impairment that may be the result of, or worsened by, sarcopenia. In 2000, the direct healthcare costs attributable to sarcopenia alone reached an estimated $18.5 billion (9, 10). Despite these risks, identifying patients for treatment remains challenging (11), in part because no uniform or generally accepted diagnostic criteria exist (12). In 2016, a revision of the International Statistical Classification of Disease introduced a code for sarcopenia (M62.84); however, it does not account for, or distinguish between, varying levels of severity. In fact, most criteria use only binary diagnostic criteria—i.e., whether a patient meets the clinical criteria for sarcopenia or not—to establish the presence of sarcopenia (2, 12, 13). A significant amount of variation in severity and appropriateness for intervention likely exists among individuals who meet the typical binary diagnostic criteria for a sarcopenia. Thus, a natural question is whether one can create a continuous measure of sarcopenia severity with an index.

There has been some progress. Others have created an index for the severity of frailty (14), an age-related condition closely related to sarcopenia, based on the number of “health deficits,” including impaired walking, comorbidities, and limitations in activities of daily living (ADLs) (15). The European Working Group on Sarcopenia in Older People defines ‘severe sarcopenia’ as reduced muscle mass, strength, and performance (2). Janssen and co-authors proposed thresholds for skeletal muscle index to distinguish individuals into two broad categories: moderate- versus high-risk of sarcopenia-related physical disability/difficulty with ADLs (13).

Few studies have evaluated the economic consequences of sarcopenia or the potential benefits of reducing the severity of sarcopenia among older individuals (10). Better understanding the societal burden of sarcopenia and the potential to reduce that burden is key to motivating policies and communicating the value of novel interventions that reduce the severity of sarcopenia. Given the large and likely growing burden of sarcopenia, the goals of this study were to develop a sarcopenia severity index and estimate the societal value generated by reducing the severity of sarcopenia in older adults over a period of 30 years in the US using a micro-simulation model.

## Methods

### Study population

The population of interest included older adults at risk for age-related muscle loss who would be good candidates for interventions aimed at reducing the development and progression of sarcopenia.

### Index development

We used a sample of older adults in the 1999–2000 and 2001–2002 National Health and Nutrition Examination Survey (NHANES) with information on muscle mass, muscle (quadriceps) strength, age, weight, and height to develop the severity index. Of 2,438 older adults in the NHANES sample, 804 (33%) were excluded due to missing information on one or more of these characteristics. The Health and Retirement Survey (HRS) 2010 wave was used to model and confirm that sarcopenia severity has a strong association with health and economic outcomes including mortality, hospitalizations, office visits, and medical expenditures.

### Simulation model

Data from two databases were used for the purposes of running the micro-simulation model. The cohort of older adults was extracted from the weighted (to be nationally representative) HRS 2010 wave. The Medicare Current Beneficiary Survey was used to estimate total medical expenditure for individuals who were age-eligible for Medicare. These expenditures were broken down by Medicare program (Parts A, B, and D; all enrollee expenses were modeled as feefor- service plans) and total versus out-of-pocket expenditures.

### Severity index component candidates

Grip strength, muscle mass (kg), appendicular lean mass (kg) adjusted for body mass index (BMI, kg/m2), sex, age, and gait speed (m/s) were considered for inclusion in the severity index. Grip strength was not available in the 1999–2002 NHANES surveys; therefore, we imputed right-hand grip strength in the NHANES sample from knee extensor/quadriceps strength based on prior literature (see supplemental material) (16). In NHANES, knee extensor strength and timed walk tests were administered in individuals ≥50 years of age without a condition/recent injury that prevented them from walking. A dynamometer was used to evaluate knee extensor strength (reported in peak torque, Newton meters). Gait speed was calculated based on the time (seconds) to complete a 20-foot walk and flagged if the respondent used an aid during the timed walk test. Body composition and lean mass were measured using dual-energy x-ray absorptiometry.

To validate the severity index components—stage one in the index development process—information on cognitive function from the NHANES sample was also used, including (i) the number of questions answered correctly on the Wechsler Adult Intelligence Scale, Third Edition, (ii) systolic blood pressure, (iii) self-reported problems with memory/confusion that creates difficulty/limitations, and (iv) self-reported difficulty with managing money. The rationale for including information on cognitive function is described in the Statistical Analysis Section, below.

In the HRS sample, all older adults without a condition or recent injury that prevented them from walking were eligible for the timed walk on a 12-foot course. Gait speed was flagged if the respondent used an aid during the timed walk test. Body weight and height were measured by trained health technicians in individuals who were able to stand and weighed <300 pounds.

### Societal value

**Figure 1:**
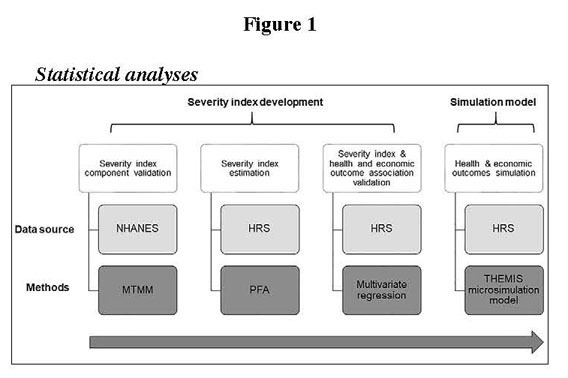
Flow diagram of articles included in the present study

Total societal benefits include changes in total medical expenditures (all inclusive, regardless of payer) for the study population and monetized quality-adjusted life years (QALYs). Medical expenses were adjusted to 2015 dollars in real terms according to the Congress Budget Office’s projections, tied to the real growth in GDP. Changes in earned income, Supplemental Security Income, and other economic outcomes were also examined, but the impacts in both intervention scenarios were small due to the demographics of the cohort of older adults, and thus were not included in the societal value. All cumulative and lifetime monetary outcomes were discounted to 2015 at a rate of 3% and reported as the net present value over the duration of the simulation in 2015 dollars. Per-period monetary outcomes were reported in 2015 dollars, but were not discounted. The study methodology is displayed in Figure 1.

### Statistical analyses

#### Index Development

We developed the index in two stages: (i) component validation, and (ii) index component weight estimation. First, multi-trait multi-methods (MTMM) and principal factor analyses were used to validate the severity index components. MTMM is a statistical approach used to assess the validity of a latent sarcopenia trait or characteristic of the patients along a continuum by evaluating this trait against a set of other distinct traits, where each trait is measured by a different physical mode of measurement. The MTMM analysis compared the ability of various combinations of index components to predict sarcopenia severity. While we were most interested in the sarcopenia severity trait, we needed at least two traits to conduct the MTMM analysis. Thus, we measured two traits— ‘sarcopenia’ and ‘cognitive functioning’—using a number of associated measures identified from the NHANES data. Cognitive functioning was conceptually distinct from muscle mass (see supplemental material). We fit three 3-method and three 4-method MTMM models to validate the sarcopenia severity index components. Model fit was assessed using the root mean square error of approximation statistic, as well as the Tucker-Lewis and Comparative Fit Indexes.

Second, based on the results of the MTMM analysis, principal factor analysis (PFA) was used to reduce the number of correlated observed variables to a small set of important independent variables, and estimate the weight of each index component and develop the index for the MTMM validated components based on the PFA estimated loading factors. Next, to validate the existence of a relationship between sarcopenia severity and health and economic outcomes, the associations between sarcopenia severity—as measured by the index—and one-year mortality, two-year mortality, and one-year inpatient hospital admissions were evaluated using multivariate logistic regression (see supplemental material).

#### Simulation model

The Health Economic Medical Innovation Simulation (THEMIS), a well established micro-simulation model (17, 18), was used to quantify the societal value generated in the US in 2010–2040 for a hypothetical reduction in sarcopenia severity in the cohort of older adults in 2010 (see supplemental material). Individuals were assigned a sarcopenia severity score based on the percentile of their severity index value, e.g., individuals in the 12th percentile had a sarcopenia severity score of 12. Thus, the lower the severity score, the more severe the sarcopenia. Simulated individuals face a likelihood of developing new health conditions, including hypertension, stroke, heart disease, lung disease, diabetes, and cancer, as a function of their risk factors, including race, education, marital status, smoking status, age, gender, and BMI, and their preexisting conditions.

Transitional probability (probit) models, derived from the HRS data, estimate likelihoods that patients develop each of these new conditions in each model cycle (2 years). The conditions are chronic and assumed to persist until death, and factor into subsequent time cycle probit models estimating risk of other conditions for a given patient. Risk factors, like age, BMI, and marital status, change each year if they are time varying. The probability of death is estimated based on the new conditions and risk factors, as are estimates of direct medical costs, functional status, and other outcomes. Patients who survive proceed to the next model cycle. The severity index value was a predictive factor for the physical function outcomes (ADL and instrumental ADL limitations, home help utilization, and mortality, among others).

Using THEMIS, we simulated the health and functional status, healthcare spending, and mortality experience of older adults starting in 2010 under two intervention scenarios: (i) a reduction in sarcopenia severity by improving gait speed by 0.1 m/s—considered a clinically significant increase in gait speed (19)—in those with gait speed under 0.8 m/s, and (ii) improved walking ability—i.e., eliminated difficulty walking in individuals who reported having some difficulty walking across a room and prevented individuals from developing difficulty walking in subsequent years. A gait speed of 0.8 m/s is the recommended cut-point for identifying sarcopenia based on an association with increased mortality and disability (11). This cut point is a midpoint between a gait speed associated with a high risk of adverse outcomes (<0.6 m/s) and a gait speed associated with low risk of adverse outcomes (>1 m/s) (20, 21). These interventions represent what would likely be an upper bound on societal value of intervening on these measures in this population.

## Results

In the NHANES sample (N=1634), the mean age was 74 years, mean BMI was 27.5 (i.e., overweight, but not obese), and 49% of the sample was female (Supplementary Table S2). Approximately two in five individuals reported participating regularly in moderate physical activity and only 3.5% of individuals used a walking aid.

The MTMM diagnostic statistics indicated that the handgrip strength measure did not perform as well as the gait speed measure in the MTMM model. While BMI and appendicular lean mass adjusted for BMI performed similarly, the latter was not available in HRS; therefore, the models using BMI were preferred because they allowed for validation and comparison with principal factor analysis using a HRS individual sample. Including the “aid used to complete the timed walk” measure— i.e., estimating the four-method models—improved the MTMM results (Table 1). Based on the model diagnostic statistics, we preferred the four-method model with gait speed as the performance measure of the sarcopenia trait, moderate physical activity as the self-reported trait measure, use of an assistive device in the timed walk as the self-reported physical trait measure, and BMI as the physical measure. We did, however, continue to consider the grip strength performance measure in the subsequent index estimation and principal factor analysis because it is easy to assess and a commonly collected performance measure used in sarcopenia diagnostic criteria.

As shown in the supplemental material, model diagnostic statistics suggested that the three-component PFA provided a more reliable measure of sarcopenia severity than the four-component PFA; therefore, we developed the severity index based on the three-component PFA results. In the fourcomponent PFA, grip strength had the second largest factor loading in the NHANES data and the smallest factor loading in the HRS data. This may stem, in part, from the fact that hand grip strength was inferred based on quadriceps strength in NHANES and lacks variance uncorrelated with performance on a quadriceps strength test. In the three-component PFA, gait speed had the largest factor loading, followed by no walk aid, and moderate physical activity in both the NHANES and HRS samples, providing evidence of cross-validation across two different population samplings. The PFA factor loadings and scoring coefficients are provided in the supplemental material. An individual’s severity index value is a function of the scoring coefficients and their gait speed, whether they participate in moderate physical activity regularly, and whether they use a walking aid. That is, the severity score equals [0.36 ×(gait speed)]+[0.22 ×1(moderate physical activity)]+[0.28 ×1(no walk aid)] where the 1 notation represents an indicator function such that, e.g., 1 (moderate physician activity) = 1 if the individual participates in regular moderate physical activity and 0 otherwise. The index was translated into percentiles, where lower percentiles/values represent more severe disease.

**Table 1:** MTMM Model Results by Number of Methods and Physical / Laboratory and Performance Sarcopenia Trait Measures

**Number of methods**	**Self-report measure**	**Self-report physical measure**	**Performance measure**	**Physical/lab measure**	**N**	RMSEA*	**CFI**	**TLI**
3	Moderate PA	_	Gait speed	BMI adjusted ALM	1636	0.074	0.934	0.801
3	Moderate PA	_	Handgrip strength	BMI adjusted ALM	Did not converge			
4	Moderate PA	No walking aid	Gait speed	BMI adjusted ALM	1634	0.062	0.905	0.823
4	Moderate PA	No walking aid	Handgrip strength	BMI adjusted ALM	Not full rank			
3	Moderate PA	¬–	Gait speed	BMI	1722	0.039	0.924	0.773
4	Moderate PA	No walking aid	Gait speed	BMI	1909	0.058	0.904	0.821

Note: MTMM = multi-trait multi-method; PA = physical activity; ALM = appendicular lean mass; BMI = body mass index; RMSEA = root mean square error of approximation; CFI = confirmatory fit index; TLI = Tucker-Lewis Index. (*) RMSEA<0.05 ideal; Source: Authors’calculations.

Among older adults in the HRS sample (N=952), each one-percentage point increase in severity index percentile (representing a decrease in sarcopenia severity) was associated with a 33% decrease in the risk of 1-year mortality, a 40% decrease in 2-year mortality, a 31% decrease in the frequency of 2-year hospital admissions, 13% decrease in the number of 2-year office visits, and 15% decrease in out-of-pocket medical expenditures (Supplementary Table S3).

**Figure 2:**
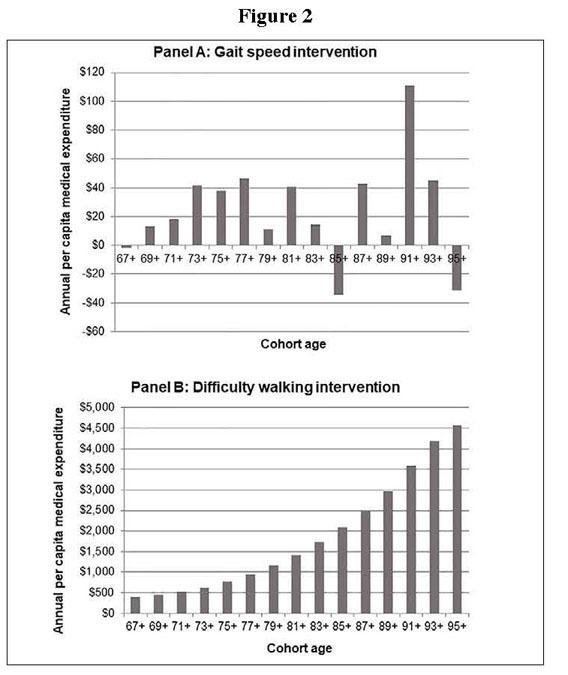
Flow diagram of articles included in the present study

Figure 2 illustrates the results of the simulation for both intervention scenarios_reducing sarcopenia severity by improving gait speed by 0.1 m/s in those with gait speed under 0.8 m/s (Panel A) and reducing sarcopenia severity by improving walking ability in those with walking difficulty (Panel B). The prevalence of difficulty with 3 or more ADLs and annual per-capita medical expenditures were not significantly reduced in the intervention to improve gait speed (Supplementary Figure S3). Neither the prevalence of difficulties with instrumental ADLs nor the frequency of transition to nursing home living were significantly reduced in either intervention scenario (see supplemental material). As we would expect, the prevalence of difficulty with 3 or more ADLs was reduced in the difficulty walking intervention, as were annual per-capita medical expenditures. The savings in annual per-capita medical expenditures in the difficulty walking intervention steadily increased in each subsequent year of the simulation—i.e., as the cohort aged—from $396 (2010 US$) in the ≥67-year-old cohort (in the first iteration of the simulation in 2012) to $4570 in the ≥95-year-old cohort (2040). Similarly, the savings in out-of-pocket medical expenditures also increased in each subsequent year of the simulation, from $50 in the ≥67 year old cohort to $799 in the ≥95 year old cohort (Supplementary Figure S4).

Cumulatively, a reduction in sarcopenia severity by improving gait speed by 0.1 m/s in those with gait speed under 0.8 m/s generated a benefit of $65B by 2040 and an average of 0.4 years of life expectancy gained per person (Table 2). A reduction in sarcopenia severity by improving walking ability in those with walking difficulty generated a cumulative benefit of $787B by 2040 and an average of 0.5 years of life expectancy gained per person (Table 2). The large difference in social value with similar life expectancy gains is due to the larger cumulative size of the cohort eligible for the walking difficulty intervention over the course of the simulation period.

**Table 2:** Social Value and Life Year Gains from Sarcopenia Severity Reduction Scenarios, 2010–2040

**Intervention**	**Social value (NPV, billions)**	**Life expectancy (years)**	**Eligible population 2010, millions (%)**
Gait speed	$43.4	0.4	4.9 (10.9)
Walking difficulty	$823.0	0.5	5.4 (12.0)

Note: NPV = net present value. Calculations assume a 3% annual discount rate 2010 US$; Source: Authors’ calculations.

## Discussion

We developed a sarcopenia severity index with three components: gait speed, participation in moderate physical activity, and no walking aid, making it a clinically tractable index of disease severity. These components are in line with previous studies that have demonstrated that participation in regular physical activity reduces the burden of major mobility disability in the elderly and that gait speed is a key predictor of mobility disability (22, 23). Gait speed thresholds were also included as measures of physical performance in several clinical diagnostic criteria for sarcopenia (2, 11, 12). As expected (3-8), we found that lower sarcopenia severity index scores, denoting more severe disease, were associated with greater odds of mortality and higher healthcare utilization. Using THEMIS, we explored the impact of two hypothetical sarcopenia severity interventions in a nationally representative cohort of older adults from 2010. The first intervention, which reduced sarcopenia severity by increasing gait speed by 0.1 m/s in those with gait speed under 0.8 m/s, generated a cumulative benefit of $65B in 2010–2040. The proportion of the cohort eligible for the second intervention (improving walking ability in those with walking difficulty) was larger and consequently so were the cumulative social benefits: $787B in 2010–2040.

This study is similar Janssen et al. (2004) study that used the share of healthcare expenditures on disability attributable to sarcopenia to estimate that the annual direct medical costs of sarcopenia in 2000 equaled $18.5B and that a 10% reduction in the prevalence of sarcopenia would save $1.1B per year [10]. Over 30 years, this is equivalent to a cumulative savings of $29.4B (2015 US$) in present value. The authors estimated that 47% of healthcare expenditures associated with sarcopenia were attributable to severe sarcopenia, defined as skeletal muscle index (muscle mass divided by height squared) ≤8.5 kg/m2 for men and ≤5.75 kg/m2 for women. Our study builds on this work in several ways. We used more recent data on healthcare expenditures, directly modeled the relationships between sarcopenia, mobility/functional disability, and health and economic outcomes, and simulated the dynamics of these relationships over time. Like Janssen et al., we demonstrate that reducing the severity of sarcopenia would generate significant health and economic benefits.

Both pharmacological and non-pharmacological treatment options for sarcopenia exist, and others are in development. Clinical trials have evaluated testosterone (in men), estrogen (in women), ghrelin, vitamin D, eicosapentaenoic acid, angiotensin-converting enzyme inhibitors, dehydroepiandrosterone, and growth hormone’s ability to prevent and/or treatment sarcopenia (24). To date, no treatment has been approved for sarcopenia. Non-pharmacological treatments are currently the mainstay of sarcopenia treatment and prevention (24, 25). Increasing and/or maintaining muscle mass through resistance exercise, protein and/or amino acid supplementation, and smoking cessation have all been demonstrated to improve muscle mass, strength, and/or gait speed and are recommended for preventing and treating sarcopenia (26-28). Maximal effectiveness—and therefore societal benefit—will likely be achieved using treatment strategies that combine both pharmacological and nonpharmacological approaches.

### Limitations

This analysis was limited by the available NHANES and HRS data. Specifically, the most recent wave of NHANES data do not include measures of muscle strength, and the biennial HRS data do not capture the short-term fluctuations in respondents’ physical performance or mobility. In addition, the simulations in this study are based on a cohort analysis (US older adults in 2010) and do not reflect the benefits of reducing sarcopenia severity overall in the future population of older adults. Given that this population is projected to grow in the coming years, the benefits of intervention in the overall population would also be larger. Lastly, the simulations assume that the improvements in gait speed or walking ability are costless. The benefits of treatments that reduce the severity of sarcopenia will have to be assessed relative to their costs.

## Conclusions

More severe deficits in a latent measure of sarcopenia are associated with increased risk of mortality and increased healthcare utilization. Reducing sarcopenia severity would likely generate significant health and economic benefits to society. Establishing diagnostic criteria and guidelines for treatment are important steps in realizing the benefits of reducing the severity of sarcopenia and will help clinicians and policymakers identify individuals most in need of treatment.

*Acknowledgements:* The authors would like to thank Michelle Brauer and Emma van Eijndhoven, employees of Precision Health Economics, for their research support.

*Ethical Standards:* This study used retrospective survey data and did not include any animal or human experiments.

*Conflict of Interest:* Dr. MacEwan reports other from Precision Health Economics, during the conduct of the study; Dr. Gill has nothing to disclose; Dr. Johnson reports personal fees from Novartis Pharmaceuticals Corporation, outside the submitted work; Dr. Doctor reports personal fees from Precision Health Economics, during the conduct of the study; personal fees from Precision Health Economics, outside the submitted work; Mr. Sullivan reports personal fees from Precision Health Economics, during the conduct of the study; personal fees from Precision Health Economics, outside the submitted work; Ms. Shim has nothing to disclose; Dr. Goldman reports other from Precision Health Economics, during the conduct of the study; other from Precision Health Economics, outside the submitted work.

## Electronic supplementary material

Multi-trait Multi-method (MTMM) Matrix Approach for Validating Sarcopenia Index Components
