# Supplementary material for: Measuring Sarcopenia Severity in Older Adults and the Value of Effective Interventions
Source: J Nutr Health Aging. 2018 Sep 18;22(10):1253–8. doi: 10.1007/s12603-018-1104-7 (PMC6302764; doi:10.1007/s12603-018-1104-7)
Supplement: Supplementary file 1 — Multi-trait Multi-method (MTMM) Matrix Approach for Validating Sarcopenia Index Components [file mmc1.pdf]

Supplemental Material

Multi-trait Multi-method (MTMM) Matrix Approach for Validating Sarcopenia Index Components

Factor analysis is a statistical method used to describe variability among observed, correlated variables in terms of a potentially lower number of unobserved variables called factors or latent variables. We used the MTMM matrix approach as our confirmatory factor analysis model in this project.

MTMM is a statistical approach to assess the construct validity of a set of measures in a study by measuring a set of *x* 'traits' or 'factors' by *y* 'methods' each. Campbell-Fiske approach to MTMM analysis is evaluating the correlation matrix to identify 3 types of correlations: homo-trait-hetero-method (same trait, different method), hetero-trait-homo-method (different trait, same method), and hetero-trait-hetero-method (different trait, different method) [1]. In this approach, the measures of the same trait should be strong (across methods), whereas different trait, same method correlations should not exceed same trait, different method correlations. MTMM is also useful as we not only validated the concept of 'sarcopenia severity', but also evaluated if this 'sarcopenia' factor is distinct relative to other factors that could affect performance (e.g., depression, cardiovascular disease, cognitive functioning).

The key advantage of the MTMM approach is assessing construct validity by demonstrating both convergent validity (i.e., whether measures that are related in theory are related in fact) and discriminant validity (i.e., whether measures that are not intended to be related are not related in fact). This is most useful for single factor validation such as that with sarcopenia. In this case, our greatest validation concern was the idea that sarcopenia measurement represents the assessment of a distinct construct and is not driven by method of measurement or strong associations with other common health concerns (e.g., cognitive functioning).

In this project, we measured two traits – 'sarcopenia' and 'cognitive functioning' – using a number of associated measures identified from the NHANES data. The measures that are associated with 'sarcopenia' are BMI adjusted appendicular lean mass, gait speed or handgrip strength, frequency of moderate physical activity or use of an assistive device to complete the timed walk. The measures that are associated with 'cognitive functioning' are cognitive test score, self-reported work limitations or difficulty managing money, and clinical hypertension (systolic and diastolic blood pressure) (see Supplementary Figure S1). Each of these measures is classified as one of three methods: 'physical measurement', 'physical performance' and 'self-report'. In the MTMM approach, a trait is measured by each of several methods by examining the correlation matrix by trait within methods. Ideally, we would measure each trait by each method.

Supplementary Figure S1 illustrates the correlations modeled in the MTMM model. The ovals indicate latent, unobservable variables ('traits'); rectangles indicate fiobserved variables ('measures') with indication of 'methods'; uni-directional arrows denote the reflective relationship between the latent trait and observed measures; bi-directional arrows denote correlations. The arrows between the latent trait and the observed measures are unidirectional and flow from the latent trait to the observed measures because the latent trait is reflected in the observed measures [2].

Supplementary Figure S1. Final Correlated Uniqueness Model in the MTMM Modeling

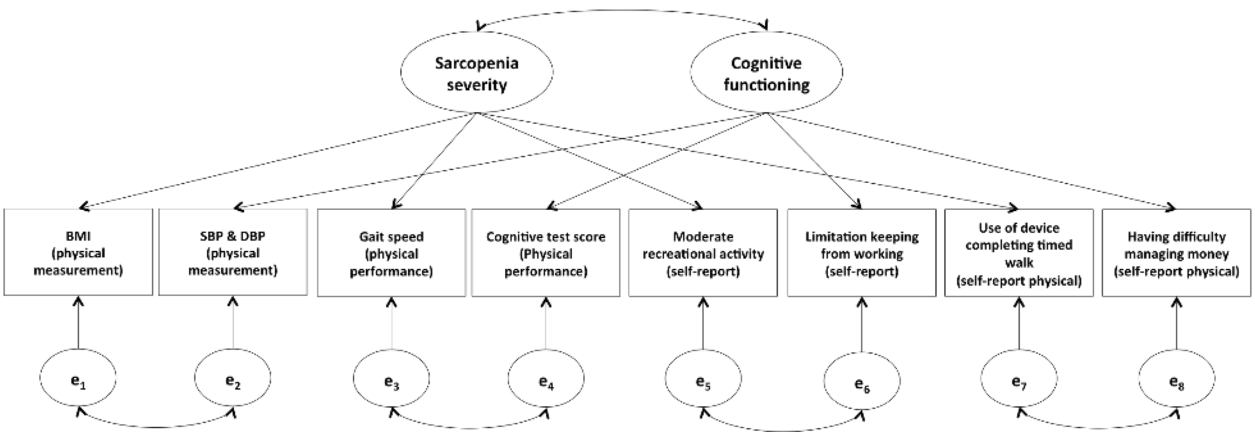

Factor Analysis and Index Estimation

Based on the MTMM analyses, we proceeded with the four validated sarcopenia index components—BMI, gait speed, self-report on moderate physical activity, and self-report on device assisted walk test—and conducted principal factor analyses (PFA) and principal component analysis (PCA) to estimate the sarcopenia severity index. We further applied PCA to reduce correlated observed variables to a small set of important independent variables and estimated weight of each measure in predicting severity score. As a validation exercise, we also compared the estimated loading factors using the NHANES patient sample versus the HRS patient sample. The Cronbach's α statistics suggest that the three-predictor model is more reliable than the four-predictor model, therefore we proceeded with the analysis using the three-predictor model.

Supplementary Table S1. Principal Factor Analysis Scoring Coefficients

Component	Factor loading	Scoring coefficient
Gait speed	0.492	0.359
Moderate PA	0.334	0.222
No walking aid	0.408	0.281

Note: Values are un-weighted. PA, physical activity.

Source: 1999–2002 National Health and Nutrition Examination Survey and authors' calculations.

Grip Strength Estimation in NHANES Sample

Grip strength was not available in the 1999–2002 NHANES surveys; therefore, we imputed grip strength in the NHANES sample from knee extensor/quadriceps strength using a published

study on the relationship between hand grip and quadriceps strength [3]. In NHANES, knee extensor strength and timed walk tests were administered in individuals ≥50 years of age who did not have a condition/recent injury that prevented them from walking. A dynamometer was used to evaluate knee extensor strength (reported in peak torque, Newton/meters).

Bohannon et al. (2012) estimated the correlation between hand grip (measured in Newtons) and knee extensor strength (measured in Newton/meters) in a sample of 164 18–85 year old individuals [3]. They found a correlation of 0.805 between both right and left hand grip strength and right knee extensor strength. Given information on the distributions (mean and variance) of hang-grip and knee extensor strength, we converted handgrip strength from Newtons to kilograms force (1kgf = 9.807N) and used this correlation to calculate the linear prediction of (right) handgrip strength (H) for a given (right) knee extensor strength (knee extension torque, Nm) (K). That is:

$$H = \beta_0 + \beta_1 K + \varepsilon.$$

Give that:

$$\mathrm{Corr}_{H,K} = \frac{\mathrm{Cov}_{H,K}}{\mathrm{se}_K \times \mathrm{se}_H},$$

and

$$\beta_1 = \frac{\mathrm{Cov}_{H,K}}{\mathrm{var}_K} = \frac{\mathrm{Cov}_{H,K}}{\mathrm{se}_K \times \mathrm{se}_K},$$

it follows that

$$\beta_1 = \frac{\mathrm{Corr}_{H,K}}{\mathrm{se}_K} \mathrm{se}_H.$$

We can also solve for

$$\beta_0 = \bar{H} - \beta_1 \bar{K}.$$

The standard error of the mean can be expressed in terms of standard deviation and sample size, i.e.,

$$SE = \frac{SD}{\sqrt{N}}$$

Based on the standard errors and sample sizes observed in Bohannon et al. (2012) we solved for:

$$SE_H = \frac{SD_H}{\sqrt{N_H}} = \frac{119.6}{\sqrt{164}} = 9.3427,$$

and

$$SE_K = \frac{SD_K}{\sqrt{N_K}} = \frac{71.8}{\sqrt{164}} = 5.6066.$$

Thus, we estimated

$$\beta_1 = \frac{0.805}{5.6066} 9.3427 = 1.34,$$

$$\beta_0 = 349.9 - 1.34 \times 171.72 = 119.8,$$

and

$$H = 119.8 + 1.34K.$$

where H = handgrip strength (N), K = knee extension torque (Nm). To translate handgrip strength in Newtons to Kgf, divide by 9.807 (1kgf = 9.807N).

Sarcopenia Severity Association with Health and Economic Outcomes

We conducted multivariable analyses to measure the associations of the sarcopenia severity index as a predictor with health care utilization (e.g., number of hospitalizations in the study period), costs (e.g., total health care costs) in the HRS data, and mortality (death within 1 or 2 years). That is, we estimated:

$$y_i = f(s_i | \boldsymbol{x_i}),$$

where y_i represents the outcome of interest (e.g., death or OOP medical expenditures), s_i represent the sarcopenia severity index percentile (score), and $\boldsymbol{x}_i$ is a vector of patient characteristics. Logistic regression was used for the mortality outcomes, Poisson regression was used for the inpatient and outpatient visit count outcomes, and generalized linear models with log link and gamma family functions were used for the OOP spending outcome. In the analyses above, we controlled for the following patient characteristics: (i) demographics (age, marital status, education, race, etc.), (ii) comorbidities (diabetes, cancer, heart disease, lung disease, etc.), and (iii) geographic region.

THEMIS Simulation Model

THEMIS, a well-established microsimulation model [4, 5], was used to estimate the long-term economic and medical costs associated with changes in the severity of sarcopenia. These costs provide a basis for ascertaining the value of treatments for sarcopenia. A module specific to sarcopenia was built within the THEMIS framework.

THEMIS is a microsimulation that tracks individuals aged over 50 through time to project their disease and comorbidity burdens including falls and fractures, life expectancy and functional status, health care costs, employment status, and personal taxes and transfer payments (including disability insurance receipts). The model was estimated using the imputed prevalence and incidence of sarcopenia. Once the sarcopenia severity has been imputed, models can be estimated and used to predict severity of sarcopenia in the future. This simulation produces long-term estimates of the societal costs of changes in the severity of sarcopenia.

THEMIS uses a number of projection techniques and sources when forecasting incoming cohorts for the model (those who age into the sampling frame in the future, about whom the HRS has no data). To get accurate forecasts, it is necessary to estimate the detailed characteristics of the future population as they enter the model, as well as the macro-economic trends they will be subject to.

A multitude of data sources are used to compute US trends in the model. First, National Health Interview Survey (NHIS) is used for chronic conditions, applying the methodology discussed in

Goldman et al. [6]. The method consists of projecting the experience of younger cohorts into the future until they reach age 51. The projection method is tailored to the synthetic cohorts observed in the NHIS. For example, a representative sample of age 35 individuals born in 1945 is observed. Their disease patterns are tracked in 1980 to 1981 surveys by then selecting those aged 36 in 1981, accounting for mortality, etc. Information on other trends (i.e., obesity and smoking) was collected from other studies [7-12].

Real growth in wages and medical costs are trended using government projections. Firstly, as is done in the Social Security Administration Trustees Report Intermediate Scenario [13], a long-term real increase in wages (earnings) of 1.1% per year is assumed. As is done by the Centers for Medicare & Medicaid Services excess real growth in medical costs (i.e., additional cost growth to GDP growth), is assumed to be reducing linearly to 1% in 2033, 0.4% in 2053, and -0.2% in 2083. Baseline medical spending figures presented assume that the Affordable Care Act cost growth targets are met. GDP growth in the near-term (through 2019) is based on Congressional Budget Office's projections, with the Old-Age, Survivors, and Disability Insurance Trustees assumption of 2% yearly afterwards [14].

For demographic trends, the model makes two adjustments to the weighting in the HRS data to match population counts from the Census. First, the HRS sample is stratified by 5 year age groups, gender, race, and rebalanced weights using the Current Population Survey (CPS). The CPS is itself matched to the decennial Census. Since some cases from the data were deleted, this step ensures that the population is still nationally representative. The second adjustment is to scale up weights for future new cohorts using population projections from the Census Bureau, again done by race and gender. The simulation uses the intermediate net migration scenario produced by Social Security Administration (SSA).

The mortality model is composed of two parts. The first part is time-invariant and relates the current state of each person to his or her probability of dying over the course of the next time period. That probability is then modified with a macro-level trend in the overall death rate, as projected by the SSA Trustees' Report [13]. This generally results in an elongation of morbidity as our status quo simulation. Recent modification of this assumption achieved the projected reductions in mortality with a tailored mix of morbidity compression and extension of the end-of-life period [15].

The model's analytic framework is able to accommodate many sources of data because of its modular nature. One of the advantages of THEMIS is that it uses real, rather than synthetic, cohorts within the simulation; even the future cohorts are based on real persons derived from the host dataset (currently the HRS). While the HRS has lots of data on health conditions, risk factors, and economic outcomes, it is by no means an exhaustive survey. When incorporating an outcome not found in the HRS, an established procedure exists for deriving the outcome in an ancillary dataset and then using a crosswalk to get that outcome into the simulation. One of the earliest examples of this is the EQ-5D, a quality-of-life measurement. While the HRS does not have the necessary survey questions to derive the EQ-5D, the information is available from the Medical Expenditure Panel Survey (MEPS). THEMIS can incorporate EQ-5D as an outcome using a model derived in the MEPS, but transferred to run in the HRS-based simulation.

Transitional probability models: How THEMIS works

Supplementary Figure S2 illustrates how THEMIS works.

Supplementary Figure S2. Illustration of THEMIS

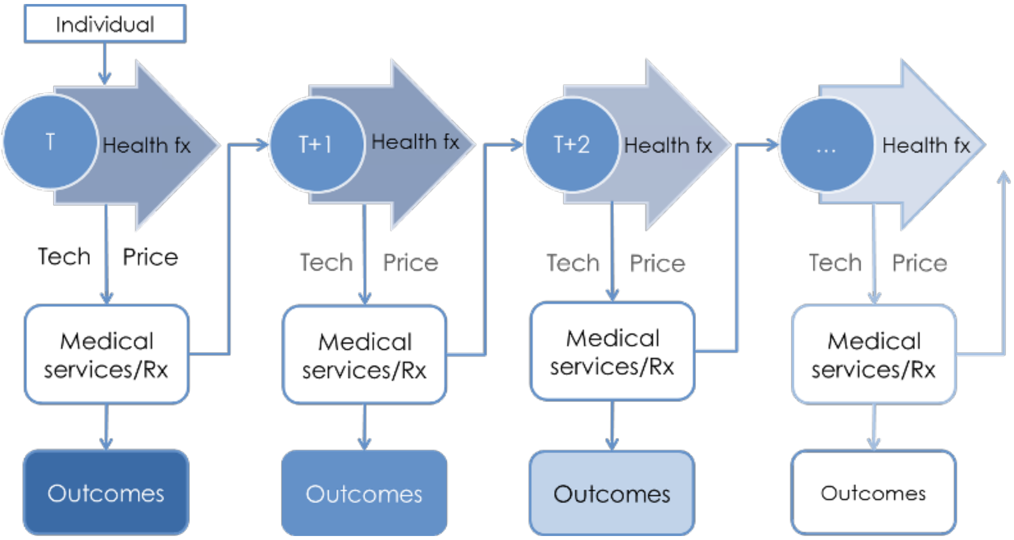

Note: "Health fx", in the blue arrows, represents the risk factors, health conditions, and survival status of a simulated individual. Each time period, a simulated individual accesses some level of technology, accrues costs, based on consumption of medical services, and health outcomes, including being alive or not and functional status translated into health utility. That individual also faces transitional probabilities in terms of acquiring new conditions and surviving, which inform his or her Health fx in the next time period.

Sarcopenia transitional probability model

To accommodate this study, THEMIS was modified to address the following research question: For the general elderly population in the US, what is the likelihood that a person gets sarcopenia? A transitional probability (probit) model of the risk of incident case of sarcopenia was developed and took the form:

$$\Pr(\text{Sarcopenia}_{it}=1|\mathbf{X}, \mathbf{\Theta}, \mathbf{T}) = \Phi(\beta_0 + \boldsymbol{\delta}\mathbf{X}_{it} + \boldsymbol{\lambda}\mathbf{\Theta}_{it} + \boldsymbol{\alpha}\mathbf{T}_t),$$

where $\mathbf{X}_{it}$ are the THEMIS risk factors and δ the change in the likelihood of sarcopenia related to a one unit change in the associated risk factor. $\mathbf{\Theta}_{it}$ represents the THEMIS conditions, and λ represents the associated effects on sarcopenia. Finally, $\mathbf{T}_t$ are the year fixed effects.

Intervention scenarios

We simulated two sarcopenia interventions using the THEMIS sarcopenia module:

- A reduction in sarcopenia severity by improving gait speed by 0.1 meters/second in those with gait speed under 0.8 meters/second. Such improvement in gait speed is considered a clinically significant increase in gait speed [16].
- A reduction in sarcopenia severity by improving walking ability in those with walking difficulty. Any respondent predicted to have difficulty walking (modeled as a binary outcome) had the limitation removed completely in this intervention scenario.

Additional Tables and Figures

Supplementary Table S2. Descriptive Statistics for Sarcopenia and Cognitive Function Trait Measures

	NHANES Sample
Observations, N	1634
Age, mean (SD)	74.0 (6.2)
Female, *n* (%)	798 (48.8)
**Sarcopenia trait**	
Gait speed (m/s), mean (SD)	0.9 (0.2)
ALM adjusted for BMI, mean (SD)	0.7 (0.2)
Moderate PA, *n* (%)	697 (42.7)
No walking aid, *n* (%)	1577 (96.5)
BMI, mean (SD)	27.5 (5.0)
**Cognitive function trait**	
Systolic BP, mean (SD)	146.7 (22.6)
Cognitive function score, mean (SD)	41.1 (17.8)
No memory problems, *n* (%)	1465 (89.7)
No problem managing money, *n* (%)	1541 (94.3)

Note: Values are un-weighted. BP, blood pressure; ALM, appendicular lean mass; BMI, body mass index, PA, physical activity. Cognitive function score equaled the number of questions answered correctly on the Wechsler Adult Intelligence Scale, Third Edition.

Source: 1999–2002 National Health and Nutrition Examination Survey and authors' calculations.

Supplementary Table S3. Estimated Relationship between Sarcopenia Severity Score and Health and Economic Outcomes

	1-yr mortality (OR)	2-yr mortality (OR)	2-yr hospital stays (IRR)	2-yr office visits (IRR)	OOP medical expenses (CR)
Sarcopenia score	0.63	0.57	0.68	0.88	0.85
	(0.47–0.83)	(0.45–0.71)	(0.62–0.75)	(0.80–0.96)	(0.77–0.94)

Notes: Odds ratios (ORs) are reported for mortality outcomes, incidence rate ratios (IRRs) are reported for health care utilization outcomes, and cost ratios (CRs) are reported for expenditure outcomes. 95% confidence intervals reported in parenthesis. Models also includes demographics, living arrangements, comorbid conditions, and region.

Odds ratios reported for the 1- and 2-year mortality outcomes, incidence rate ratios reported for the hospital stays and office visit outcomes, and cost ratios reported for the OOP medical expenses outcome. All results significant at $p < 0.001$.

Source: 2010 HRS and authors' calculations.

Supplementary Figure S3. Prevalence of Difficulties with Activities of Daily Living for Sarcopenia Severity Intervention Focused on Walking Difficulty

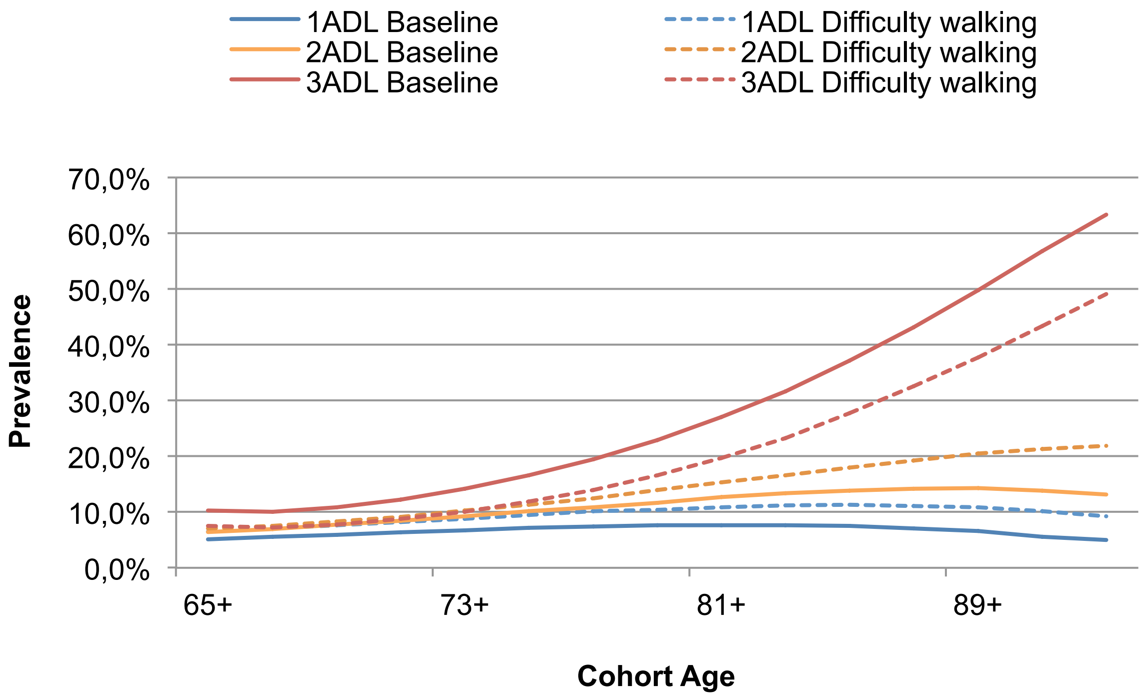

Supplementary Figure S4. Change in Out-of-Pocket Per Capita Medical Spending by Sarcopenia Intervention Scenario

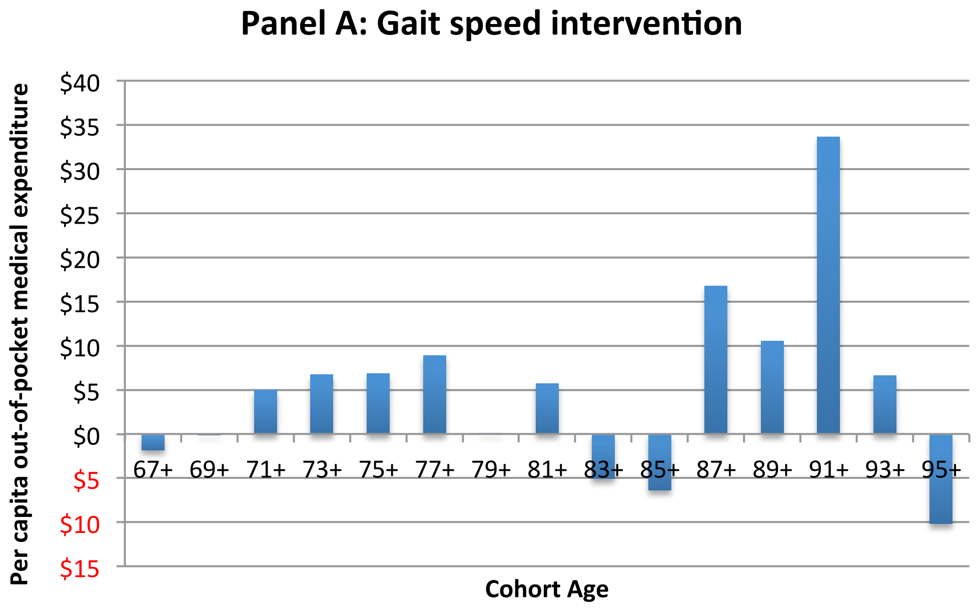

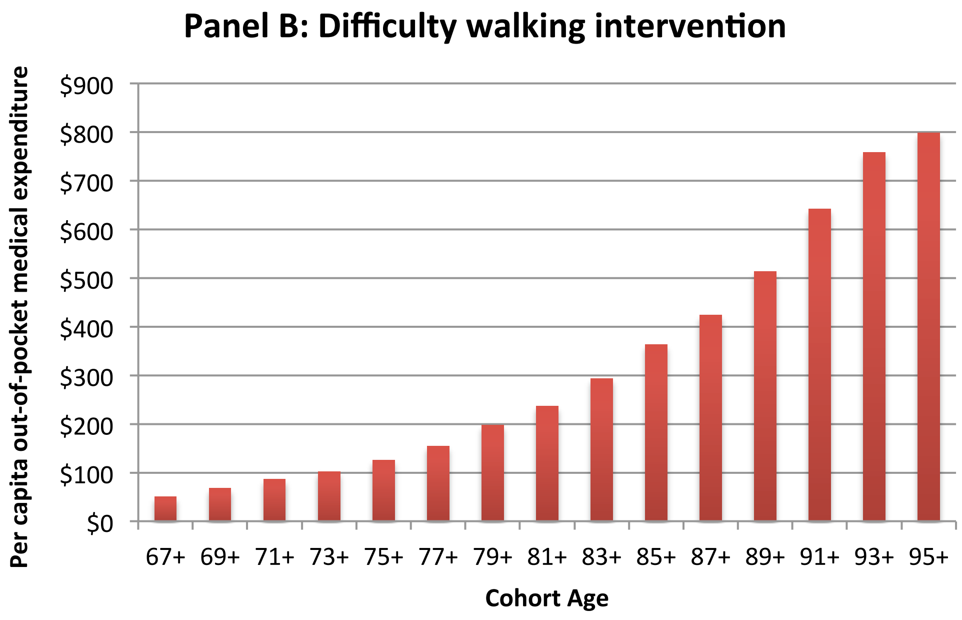

Supplementary Figure S5. Prevalence of Any Difficulty with Instrumental Activities of Daily Living by Sarcopenia Intervention Scenario

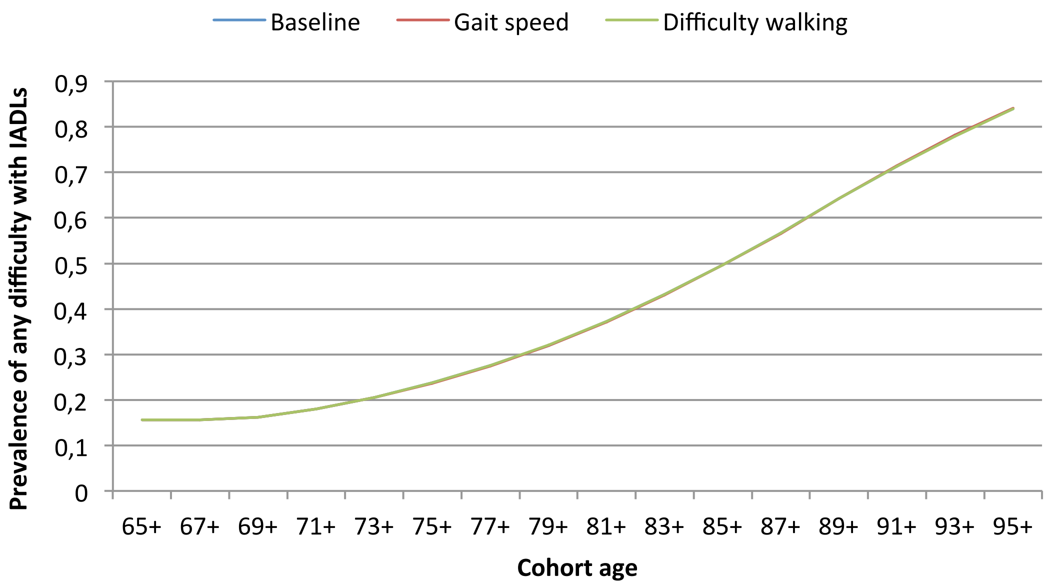

Supplementary Figure S6. Prevalence of Nursing Home Living by Sarcopenia Intervention Scenario

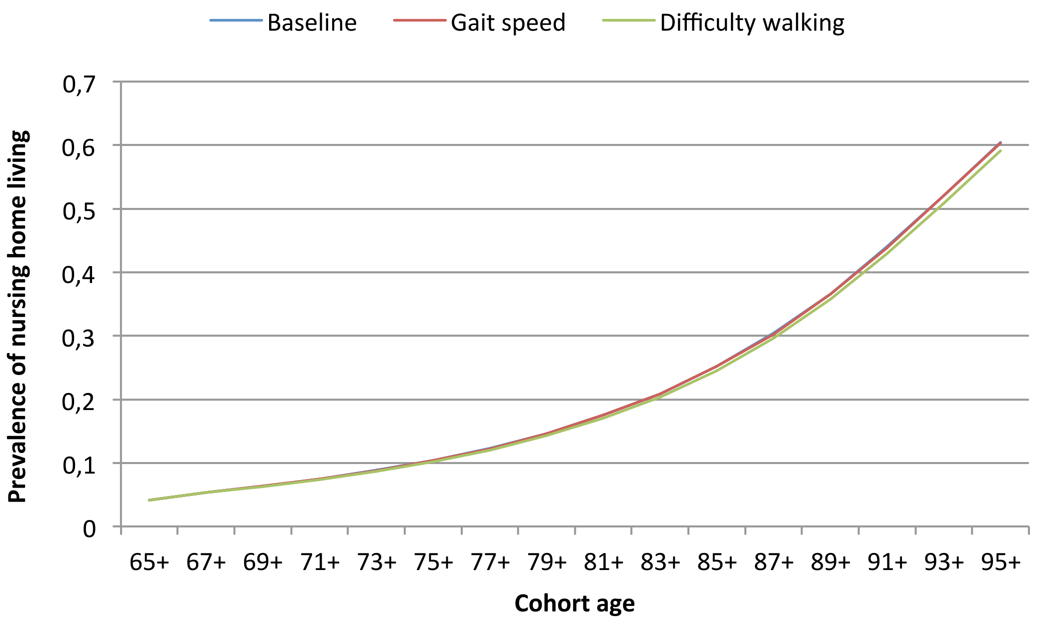

References

1. Campbell, D.T. and D.W. Fiske, *Convergent and discriminant validation by the multitrait-multimethod matrix.* Psychological bulletin, 1959. **56**(2): p. 81.
2. Coltman, T., et al., *Formative versus reflective measurement models: Two applications of formative measurement.* Journal of Business Research, 2008. **61**(12): p. 1250-1262.
3. Bohannon, R.W., et al., *Grip and knee extension muscle strength reflect a common construct among adults.* Muscle & nerve, 2012. **46**(4): p. 555-558.
4. Lakdawalla, D., et al., *Measuring the value of better diabetes management.* The American journal of managed care, 2013. **19**: p. E11.
5. Goldman, D.P., et al., *Substantial health and economic returns from delayed aging may warrant a new focus for medical research.* Health affairs, 2013. **32**(10): p. 1698-1705.
6. Goldman, D., et al., *Health status and medical treatment of the future elderly: Final Report*. 2004, RAND Corporation: Santa Monica, CA.
7. Honeycutt, A.A., et al., *A dynamic Markov model for forecasting diabetes prevalence in the United States through 2050.* Health care management science, 2003. **6**(3): p. 155-64.
8. Levy, D., *Trends in Smoking Rates Under Different Tobacco Control Policies:Results from the SimSmoke Tobacco Policy Simulation Model*. 2006.
9. Poterba, J., S. Venti, and D.A. Wise, *The Decline of Defined Benefit Retirement Plans and Asset Flows*. 2007, National Bureau of Economic Research, Inc, NBER Working Papers: 12834.
10. Poterba, J., S. Venti, and D.A. Wise, *New Estimates of the Future Path of 401(k) Assets*. 2007, National Bureau of Economic Research, Inc, NBER Working Papers: 13083.
11. Ruhm, C., *Current and Future Prevalence of Obesity and Severe Obesity in the United States.* Forum for Health Economics & Policy, 2007. **10**(2).
12. Mainous, A.G., 3rd, et al., *Impact of the population at risk of diabetes on projections of diabetes burden in the United States: an epidemic on the way.* Diabetologia, 2007. **50**(5): p. 934-40.
13. Online, S.S., *Table V.A1.- Principal Demographic Assumptions, Calendar Years 1940-2085*. 2009.
14. Social Security Administration. *The 2016 Annual Report of the Board of Trustees of the Federal Old-Age and Survivors Insurance and Federal Disability Insurance Trust Funds*. 2016; Available from: https://www.ssa.gov/OACT/TR/2016/II_C_assump.html#95492.
15. Clark, M.K. and J.S. Dillon, *BMI Misclassification, Leptin, C-Reactive Protein, and Interleukin-6 in Young Women with Differing Levels of Lean and Fat Mass.* Obesity Research & Clinical Practice, 2011. **5**(2): p. e85-e92.
16. Perera, S., et al., *Meaningful change and responsiveness in common physical performance measures in older adults.* Journal of the American Geriatrics Society, 2006. **54**(5): p. 743-749.
